# Effect of surface dielectric barrier discharge plasma on the physicochemical properties of soy protein isolate-chia seed gum complex

**DOI:** 10.1016/j.fochx.2025.102574

**Published:** 2025-05-22

**Authors:** Soheila Ahmadian, Farshad Sohbatzadeh, Fatemeh Jamshidi Alashti, Reza Esmaeilzadeh Kenari

**Affiliations:** a Department of Food Science and Technology, Faculty of Agricultural Engineering, Sari Agricultural Sciences and Natural Resources University, Km 9 Farah Abad Road, Sari, Iran; b Department of Atomic and Molecular Physics, Faculty of Science, University of Mazandaran, Babolsar, Iran

**Keywords:** Cold atmospheric plasma, FTIR, Functional properties, Rheological properties, Surface hydrophilicity

## Abstract

The aim of this study was to modify the soy protein isolate-chia seed gum (SPI-CSG) complex using surface dielectric barrier discharge plasma at different exposure times (2, 4, and 6 min) to improve its techno-functional potential. The present active species in the plasma were determined using optical emission spectroscopy. The results revealed that the surface properties of the complex were altered from hydrophilic to hydrophobic at 4 min treatment and then reverted to hydrophilic at 6 min due to polar group formation. The apparent viscosity and modulus decreased with increasing plasma exposure time. FTIR analysis confirmed that plasma treatment did not alter the main structure of the complex, although the intensity of the peaks changed. The main driving forces for the SPI-CSG complex formation were hydrogen bonding, hydrophobic, and electrostatic interactions. Moreover, the plasma-treated solution exhibited a reduction in particle size and increased antioxidant activity compared to the untreated solution.

## Highlights

• FTIR analysis showed cold plasma (CP) changed the intensity of the peaks.
• CP altered surface hydrophilicity of soy protein isolate-chia seed gum (SPI-CSG) complex.
• Plasma treatment modified the rheological properties of the SPI-CSG complex.
• Plasma exposure increasing up to 6 min reduced particle size of the SPI-CSG complex.

## 1 Introduction

The main components of structured food design are proteins, polysaccharides, and their combination. They transfer specific functional qualities, like gelling and emulsifying agents, to alter rheological features (Ge et al., 2021). In food systems, the protein-polysaccharide interaction is crucial for stabilizing colloidal systems, especially emulsion-based delivery systems. Indeed, in the creation of stable colloidal food formulations, these biopolymeric interactions combine the emulsifying capacity of protein with the stabilizing influence of polysaccharides (Boonlao et al., 2020).

Much current research in this field focuses on the particular case of plant proteins regarding the trend toward using more sustainable protein sources. In addition to being used for food structure, plant proteins and polysaccharides are mixed for non-food purposes such as packaging films and fabric fibers. Many plant proteins are less hydrophilic and frequently cannot be readily extracted from the raw plant material in its native state compared to the extensively researched soluble globular animal proteins, such as milk and egg proteins. This creates a new set of challenges and opportunities when investigating protein-polysaccharide interactions (Li & de Vries, 2018). Soy protein isolate (SPI) is a vegetable protein with the same amino acid composition as milk protein, and it has high nutritional value and functional quality. Soy products contain beneficial chemicals, such as soy isoflavones, that may protect against cardiovascular illnesses (Ahmadian et al., 2024; Q. Cui et al., 2020). However, SPI faces challenges such as low solubility, inadequate emulsification, and sensitivity to pH, ionic strength, and temperature, whether due to inherent properties or processing methods, which restrict their usage. For this reason, the functional properties of SPI are frequently enhanced through physical, chemical, and biological modifications to satisfy the requirements for particular functions in various areas (Li et al., 2023; Tan et al., 2024).

Since mucilages have a high water-holding capacity and a distinct ability to form a gelatinous mass when soaked in water, using them as emulsifiers and stabilizers for emulsions is a novel idea (Hernández-Nava et al., 2020). Chia seed gum (CSG) is an anionic heteropolysaccharide that has emerged as a novel hydrocolloid source for the food industry due to its biodegradability and biocompatibility nature. When the seed is exposed to water, it exudes and has a high viscosity even at low concentrations. The high fiber and carbohydrate content of CSG enhances its encapsulating ability, as it tends to form a gel, while its high protein content improves its emulsifying capacity (Cortés-Camargo et al., 2019; de Campo et al., 2017). According to studies, the impacts of environmental stress can be decreased by employing proteins coated with polysaccharides as emulsifiers. Moreover, adding polysaccharides can enhance the functionality of proteins (F. Cui et al., 2021; Yan et al., 2020).

It is well acknowledged that biopolymer modification is an essential method for altering the physicochemical properties of materials, leading to the creation of new materials with enhanced properties and enabling them to be explored to their full potential in many application areas (Das et al., 2023).

Cold plasma is an innovative technology that has gained attention for modifying food components (Jiang et al., 2020). The potential of cold plasma treatment as a promising alternative to traditional methods for modifying food biopolymers has been highlighted in literature due to its safety, simplicity, eco-friendliness, effectiveness, and profitability. The alteration is primarily induced by beginning and promoting a variety of potential chemical reactions in the plasma environment, including cleavage, oxidation, polymerization, and cross-linking reactions. These reactions occur via active species interacting with food-constituent macromolecules in the plasma media (Sadeghi et al., 2021). Cold plasma comprises a diverse array of reactive species, including free radicals, excited atoms and molecules, negative and positive ions, electrons, and UV radiations, which makes it effective in food processing (Jiang et al., 2020). Plasma-active species can be delivered to the sample directly or indirectly, as well as via plasma-activated solutions such as water. In the direct exposure method, the food comes into direct contact with the plasma discharge, allowing maximum interaction between the food and reactive species. However, achieving uniform treatment on complex surfaces with pores can be challenging. Indirect exposure involves the transfer of generated plasma through the input gas flow onto the surface, allowing the treatment surface to be positioned separately from the plasma-generating electrodes. It is crucial to note that only relatively long-lived species can reach and interact with the target because higher chemical activity means a shorter lifetime for the active species. Reactive species can be transported more uniformly and negative consequences are prevented, even though lower activity is attained than direct exposure (Kopuk et al., 2022). Tan et al. (2024) reported that treating SPI with corona discharge cold plasma at 14 kV for 90 min enhanced the surface hydrophobicity, sulfhydryl content, emulsification performance, and stability, due to the unfolding and disordering of the protein structure. Mutlu et al. (2023) found that the application of atmospheric pressure cold plasma jet to Chia seeds increased the viscosity and elasticity of chia mucilage gels by cross-linking and strengthening the polymer network, potentially opening up new food application opportunities for this gum.

Since cold plasma shows promising potential for modifying biopolymers, the aim of this study was to evaluate the effects of different exposure times (2, 4, and 6 min) of cold atmospheric plasma (CAP) treatment on the physicochemical properties of soy protein isolate-chia seed gum (SPI-CSG) complex solution. This complex was treated at a surface dielectric barrier discharge plasma in ambient air. The active species and radicals in the plasma medium were analyzed through optical emission spectroscopy.

## 2 Materials and methods

### 2.1 Materials

SPI (protein ≥90.0 %, moisture ≤7.0 %, ash ≤6.0 %, fat ≤1.0 %) was obtained by Yuwang Co., LTD. (Shandong, China). Chia seeds were provided from the local markets of Mashhad, Iran.

All chemicals were purchased from Sigma-Aldrich Co. (Tehran, Iran).

### 2.2 Extraction of CSG

Gum was extracted using the method described by Antigo et al. (2020) with some modifications. To induce mucilage exudation, chia seeds were soaked in distilled water at a ratio of 1:20 (*w*/*v*) and continuously stirred for 30 min at 50 °C. A cotton cloth (Grade 90, 44 × 36 threads per square inch) was used to remove the seeds from the aqueous suspension. After centrifuging (15 min at 3000×*g* and 25 °C - Orum Tadjhiz Co., Iran) the resulting mucilage was dried overnight at 50 °C in a conventional oven with forced air circulation.

### 2.3 Biopolymer solution preparation

CSG (0.2 g/100 mL of water) and SPI (6 g/100 mL of water) solutions were prepared in distilled water at pH 7.0. After agitation with a magnetic stirrer for 2 h at room temperature, the solutions were refrigerated overnight to achieve complete hydration. The SPI-CSG complex solution was made by combining the CSG and SPI solutions in a 1:1 (*V*/V) ratio. The mixture was re-stirred for 2 h at room temperature and then kept at 4 °C overnight. Solutions also included sodium azide (0.01 g/100 mL), an antimicrobial agent (Ahmadian et al., 2024).

### 2.4 Plasma processing

**Fig. 1:**
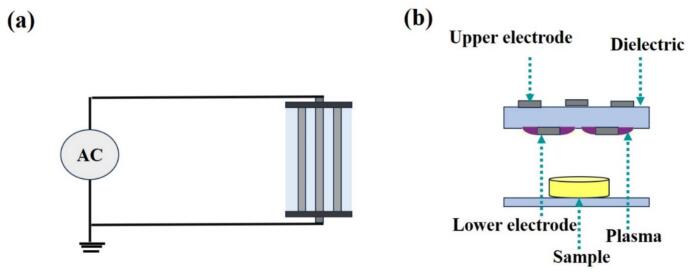
Schematic diagram of (a) top and (b) front of cold plasma set-up.

Fig. 1 exhibits a schematic diagram of the surface dielectric barrier discharge (SDBD) apparatus. The SDBD device consists of aluminum adhesive tape electrodes (2 cm × 18 cm and spaced 2 cm apart) glued edge to edge on the top and back surfaces of a glass dielectric (4 mm thick). 25 mL of SPI-CSG complex solutions were spread homogenously into glass Petri dishes (200 mm × 30 mm). The frequency and AC voltage were adjusted at 6.2 kHz and 16 kV, respectively, with atmospheric air used as the inducer gas for a treatment time of 2, 4, and 6 min. Control and plasma-treated samples were kept at 4 °C before analysis.

### 2.5 Optical emission spectroscopy (OES)

The generation of active species in the plasma and the emission lines were identified using OES. Optical emission spectra were obtained with a compact wide-range spectrometer (S100, Solar LS, Minsk, Belarus). The light emitted by the plasma is captured through quartz fiber and directed to a charge-coupled device detector for further analysis. The spectral lines were identified and smoothed using Origin 9.0 software (OriginLab Co., Northampton, USA).

### 2.6 Water contact angle measurements

The hydrophilicity of freeze-dried treated and untreated complex solutions (SPI-CSG) was evaluated by contact angle measurements. This was applied by the sessile drop method. 8 μL of distilled water was placed on the surface of the samples. A digital microscope (Dino-Lite, AM413, China) connected to a computer captured the shape of a deionized water droplet on the surface.

### 2.7 Fourier-transform infrared (FTIR) spectroscopy

The FTIR spectrophotometer (Agilent's Cary 630, Santa Clara, USA) was used to analyze the structural composition of freeze-dried treated and untreated complex solution (SPI-CSG). The powders were mixed with potassium bromide (KBr) and compressed to form pellets. Spectra were collected in transmission mode across a range of 4000–400 cm^−1^ with a resolution of 4 cm^−1^.

### 2.8 Analysis of particle size, polydispersity, and zeta potential

A dynamic light scattering method (S3500, Microtrac, USA) was employed to determine the mean particle size, polydispersity index, and zeta potential of the treated and untreated complex solutions (SPI-CSG). To reduce the impact of multiple scattering, the solutions were diluted (1:100) with deionized water. The average of three readings was given, and all tests were conducted at 25 °C.

### 2.9 Rheological properties

The rheological properties of the solutions were performed following the procedure by Huang et al. (2019) with some modifications. The analysis was conducted at 25 °C using a rheometer MCR 301 (Anton Paar GmbH, Stuttgart, Germany), equipped with a parallel plate (1 mm plate gap). Viscosity was determined by investigating the flow behavior over a shear rate range of 0.01 to 1000 s^−1^. The data was presented by plotting apparent viscosity against shear rate on a logarithmic scale. The linear viscoelastic region (LVR) of solutions was determined by conducting an amplitude sweep ranging from 0.1 % to 1000 % strain at a frequency of 1 Hz. Subsequently, their viscoelastic properties were evaluated through a frequency sweep test within the LVR region, using a strain level of 4 % and frequencies ranging from 0.1 to 100 Hz. The storage modulus (G') and loss modulus (G") values were then plotted against frequency to analyze their behavior.

### 2.10 Antioxidant activity

The capacity of solutions to scavenge DPPH free radicals was evaluated using the method described by Sarikurkcu et al. (2020). 1 mL of sample solution was mixed with 4 mL of DPPH methanol solution (0.004 g/100 mL). The sample absorbance was read at 517 nm following 30 min of incubation in the dark at room temperature. The following equation was used to calculate the DPPH radical scavenging activity.

$$\text{DPPH radical scavenging ability}\;\left(\%\right)=\left(X_{0}-X\right)/X_{0}\times 100\%$$

Where X_0_ represents the absorbance of methanol and DPPH radicals, and X represents the absorbance of extract and DPPH radicals.

### 2.11 Statistical analysis

Data were reported as means ± standard deviations. One-way analysis of variance (ANOVA) using SPSS 19.0 was used to compare differences between groups. *P*-value <0.05 was considered statistically significant by Duncan's multiple range test.

## 3 Results and discussion

### 3.1 OES analysis

**Fig. 2:**
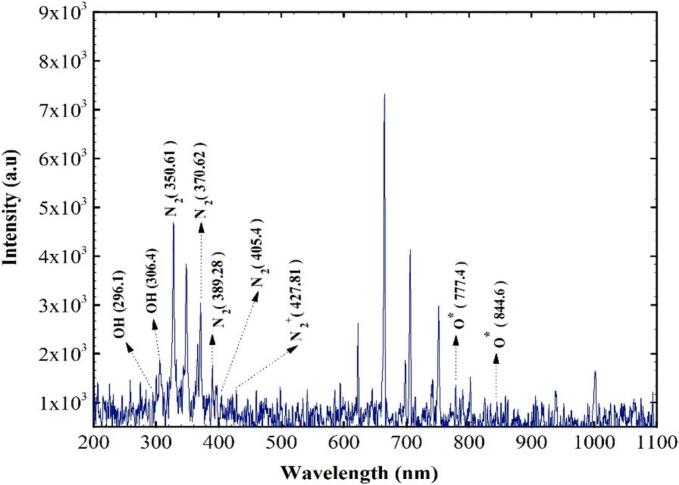
The optical emission spectrum of cold plasma.

OES is an excellent tool for investigating the excited species in plasma discharge. Fig. 2 exhibits the range of wavelengths (200 to 1100 nm) and corresponding spectra of species produced by air SDBD plasma. The spectrum showed clear peaks that correspond to different atomic and molecular transitions, signifying the presence of various reactive species within the plasma. These species play a crucial role in the plasma's chemical reactivity. The wavelengths of 296.1 nm and 306.4 nm are related to the emission lines of OH radicals (A^2^Σ → X^2^Π) (Misra, Keener, et al., 2015). Moreover, in electron impact excitation and ionization from the molecular ground state N_2_ (X^1^Σ_g_^+^), the first negative system of N_2_^+^ and the second positive system of N_2_ are frequently produced. The second positive system N_2_ (SPS) was observed in the range of 313 nm and 390 nm (Machala et al., 2007). Also, the bands between 390 nm to 450 nm are attributed to the first negative system N_2_^+^ (FNS) (Qayyum et al., 2005). The air plasma exhibits the electron transition of oxygen atoms O* from the 3p^5^P → 3s^5^P line at 777.4 nm. The 3p^5^P → 3s^3^S at 844.6 nm (X. Zhang et al., 2012). The peaks observed in this study are similar to those identified in the research by Alashti et al. (2024).

### 3.2 Surface wettability

**Fig. 3:**
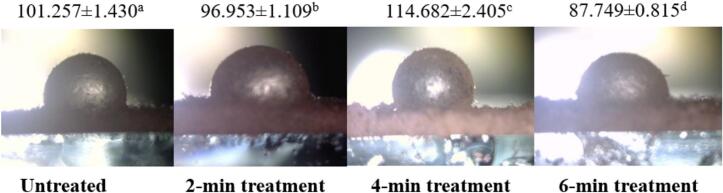
The contact angle of the control, and plasma-treated complex of soy protein isolate-chia seed gum at different times (2, 4, and 6 min).

Wettability is measured by the water contact angle. This crucial indicator can determine the hydrophilicity or hydrophobicity of materials (Basak & Annapure, 2022). Fig. 3 depicts the impact of SDBD plasma on the wettability of the SPI-CSG complex. Plasma treatment for 2 min decreased the contact angle of the biopolymer complex from 101.257 ± 1.430 to 96.953 ± 1.109. The excited species of the plasma generate polar functional groups, particularly oxygen-containing groups, which contribute to increased hydrophilicity (Dong et al., 2018). As the OES results showed, the use of air gas as the working gas led to the generation of reactive oxygen species (ROS) such as hydroxyl radicals and atomic oxygen, which may react with the surface hydrogen compound of the SPI-CSG complex and increase surface hydrophilicity (Chen et al., 2019). Results of Bormashenko et al. (2021) showed increased hydrophilicity of the SPI and milk protein concentrate powders following CAP treatment. After 4 min of plasma treatment, the contact angle increased to 114.682 ± 2.405, plasma exposure destroyed the existing polar functional groups because these groups are unstable. Indeed, short-duration plasma treatment has the potential to oxidize or introduce new hydrophilic groups onto the protein surface, which frequently bury or expose the hydrophilic groups. Nevertheless, a longer duration may cause the hydrophobic groups to surface and become exposed to a polar environment (Basak & Annapure, 2022). The contact angle was again decreased after exposing the sample to 6 min treatment. An increase in the polar anchors, particularly the hydroxyl groups shown in the FT-IR spectrum, may cause this reduction in contact angle resulting from longer treatment times. Q. Zhang, Cheng, et al. (2021) found that plasma treatment at 100 kHz for 2 min decreased the surface hydrophobicity of soybean protein isolates. With plasma exposure increasing up to 5 min, the surface hydrophobicity increased, and it again showed a decreasing trend up to 10 min.

Solid particles exhibit hydrophilicity when the contact angle is less than 90^°^ and are apt to form the O/W emulsion. Conversely, they show hydrophobicity when the contact angle is higher than 90^°^ and are suitable for preparing W/O emulsion (Yue et al., 2022). Results showed that contact angles of 6 min plasma-treated samples are less than 90°, indicating a tendency toward O/W emulsion formation. However, the 2 and 4 min treated samples are higher than 90°, potentially making them more suitable for W/O emulsion preparation.

### 3.3 FTIR spectroscopy

**Fig. 4:**
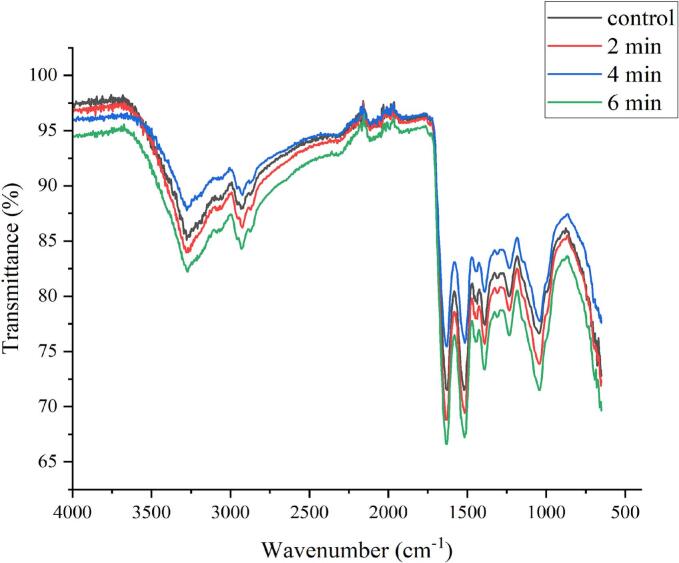
FTIR spectra of control, and plasma-treated complex of soy protein isolate-chia seed gum at different times (2, 4, and 6 min).

According to the results of our previous study, since the SPI-CSG complex included a high concentration of protein, this complex displayed bands where SPI functional groups dominated. The intensity of these bands was lower than the single SPI polymer due to an electrostatic interaction between CSG and SPI (Ahmadian et al., 2024). The FTIR spectra of the samples treated with plasma at different times (2, 4, and 6) are shown in Fig. 4. According to the results, the main structure of the complex biopolymers remained unchanged after cold plasma treatment, although there were differences in the intensity of the peaks. Bulbul et al. (2019) also showed that plasma treatment did not change the main backbone chains of xanthan gum, however, some peaks displayed a slight difference in intensity.

Our findings revealed a band at around 3271 cm^−1^, attributed to the stretching vibration of OH groups. According to the influence of plasma treatment time, the intensity of this peak increased at 2 and 6 min, demonstrating more hydroxyl groups and hydrogen bonding in the plasma-treated samples compared with the untreated sample (Yu et al., 2021), however, the intensity of the band decreased after 4 min of treatment. This indicates the loss of hydroxyl groups, which can be caused by oxidation or cross-linking (Sadeghi et al., 2021). The results of the contact angle test also showed an increase in the hydrophobicity of the sample after 4 min plasma treatment, while the 2 and 6 min treatments caused an increase in hydrophilicity. Cold plasma-generated reactive species can change the structure of proteins and expose hydrophobic amino acid residues to the surface, increasing their binding accessibility (Kopuk et al., 2022). After exposure to 2 and 6 min plasma treatment, the strength of the absorption peaks at amide I (1629 cm^−1^, C=O stretching vibration) and amide II (1517 cm^−1^, N—H bending vibration) was increased, indicating a modification in the SPI secondary structure (Zhao et al., 2021). This shows an increase in C=O content. Carbonyl groups are formed when reactive species like ROS break C—N and C—C bonds. Moreover, cold plasma treatment can generate C=O by oxidizing the side chains of amino acids containing –NH or –NH_2_ groups. The rise in N—H peak intensity is attributed to the reformation of N—H bonds caused by oxidative conditions during plasma treatment (Tan et al., 2024).

In the 4 min plasma-treated sample, the intensity of amide I and amide II decreased. Reduction in amide I intensity during plasma treatment is associated with the hydroxylation reaction that transforms carbonyl groups to carboxylic acids. In the interaction between alcohol and carboxylic acid groups, the carbonyl acts as an intermediate component (Sharafodin & Soltanizadeh, 2022). Amide II peak intensity changes might be related to the NH group's hydrogen‑deuterium exchanges, which had previously been concealed within the hydrophobic sections of protein molecules (Ghobadi et al., 2021). S. Zhang, Huang, et al. (2021) found that pea protein concentrate (PPC) exhibits an increase in absorption intensity following CAP treatment, although it still displays all the major secondary structural components. This finding suggests that the PPC secondary structures have some reorganization. Indeed, comparing the spectra of 2, 6, and 4 min treated samples showed the peaks for amide I and amide II that were higher than the 4 min treated sample. The possible explanation for this result is that during the 4 min treatment, there were more hydrophobic interactions between the SPI and CSG molecules. According to the findings of S. Chen, Li, et al. (2020), the presence of hydrophobic interaction between polysaccharides and protein is confirmed by the reduction in amides I and II. Our results demonstrated that the SPI-CSG complex formation was mainly attributed to hydrogen bonding and hydrophobic and electrostatic interactions. As a result of cold plasma treatment, the interaction within the SPI-CSG complex is strengthened, leading to enhanced performance and increased suitability as a delivery wall material.

### 3.4 Mean particle size, zeta potential, and polydispersity index (PDI)

Particle size is an essential indicator of the SPI-CSG complex's functional characteristics, and the particle size distribution of the complex particles in the solution is assessed using PDI (Wang et al., 2021). The effects of CAP treatment on the particle size, zeta potential, and PDI of the SPI-CSG complex are presented in Table 1. The average particle size of the complexes decreased significantly with increasing plasma exposure time due to the dispersion and rearrangement caused by the physical collision of high-energy CAP species (G. Chen, Dong, et al., 2020). The polydispersity index of the untreated SPI-CSG complex was 0.688 ± 0.096 and increased to 1.135 ± 0.01 by treatment time increasing up to 6 min, which means that the treated SPI-CSG complexes had more inhomogeneous mass distribution than the untreated. This result shows that after plasma treatment, more degradation occurred due to plasma free radicals. Depending on the intensity of the plasma treatment, one of the two competing reactions of depolymerization and molecular chain linking may occur in the process. Since there are more oxidizing reactive species at longer exposure times, the degradation effect can frequently be greater (Sadeghi et al., 2021). Based on the results of Momeni et al. (2018), plasma-treated pectin showed a lower molecular weight and a higher polydispersity index than the untreated sample, which may be because plasma breaks side chains that contain individual, linear, or branched residues of sugar. The net charge on the complex particles' surface is determined by the zeta potential, which is commonly utilized to evaluate the stability of the solution system. Stronger repulsion and less aggregation proneness between molecules are associated with higher absolute values of the zeta potential in the solution system (Wang et al., 2021). Our results showed that with increasing plasma exposure time, the zeta potential of the solutions indicated a slight decreasing trend. This suggested that the stability of solutions remained unchanged following plasma exposure.

**Table 1:** Particle size, zeta potential, polydispersity index (PDI), and antioxidant activity of plasma-treated soy protein isolate-chia seed gum complex solutions.

Treatment	Particle size (nm)	Zeta potential (mV)	PDI	Antioxidant activity (%)
Control	1279 ± 75^a^	−31.40 ± 0.45^a^	0.68 ± 0.09^a^	5.31 ± 0.20^a^
2 min	937.33 ± 43.24^b^	−31.06 ± 0.66^ab^	1.00 ± 0.01^b^	7.53 ± 0.31^b^
4 min	830 ± 32.41^c^	−30.36 ± 0.41^bc^	1.05 ± 0.01^bc^	10.25 ± 0.13^c^
6 min	624 ± 86.37^d^	−30.13 ± 0.25^c^	1.13 ± 0.01^c^	14.20 ± 0.62^d^

Data are expressed as the mean ± standard deviation (*n* = 3). Different letters in the same column were significantly different (*p* < 0.05).

### 3.5 Rheological properties

**Fig. 5:**
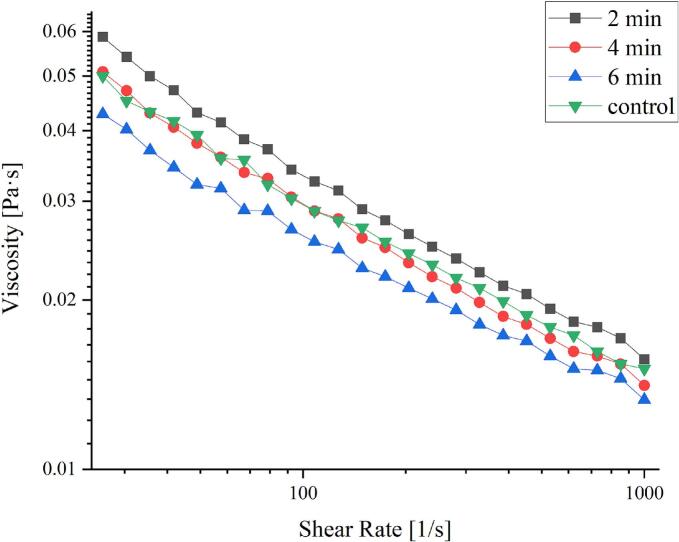
Apparent viscosity of control, and plasma-treated complex of soy protein isolate-chia seed gum at different times (2, 4, and 6 min).

Since rheological characteristics are crucial for enhancing food quality, the rheological properties of the SPI-CSG complex solutions were investigated (Gong et al., 2023). The effect of the plasma treatment on the apparent viscosity of specimens is depicted in Fig. 5. Apparent viscosity of all SPI-CSG complex solutions decreased with increasing shear rate (0.01 to 1000 s^−1^), which indicates shear thinning behavior (pseudoplastic materials). Shear thinning behavior observed in the biopolymer molecules may be explained by their orientation in the flow direction as a result of the intermolecular bond breakdown, which depends on the shear rate (Razi, Motamedzadegan, Shahidi, & Rashidinejad, 2020). In fact, the bonds between the molecules (likely hydrogen and hydrophobic binding) and the network developed around the particles break as the shear rate increases, leading to a decrease in viscosity (Amirabadi et al., 2021). In plasma-treated samples, viscosity decreased as exposure time increased due to molecular weight reduction as a result of the fragmentation of the macromolecular structure. The results of Sadeghi et al. (2021) also showed that with the increase in plasma treatment time on *Lepidium perfoliatum* seed gum, the viscosity decreased. The experimental data were well-fitted by the Ostwald-de-Waele model (R_2_ ≥ 0.9). The *n* values of all solutions were less than 1.0, indicating the shear-thinning behavior.

**Fig. 6:**
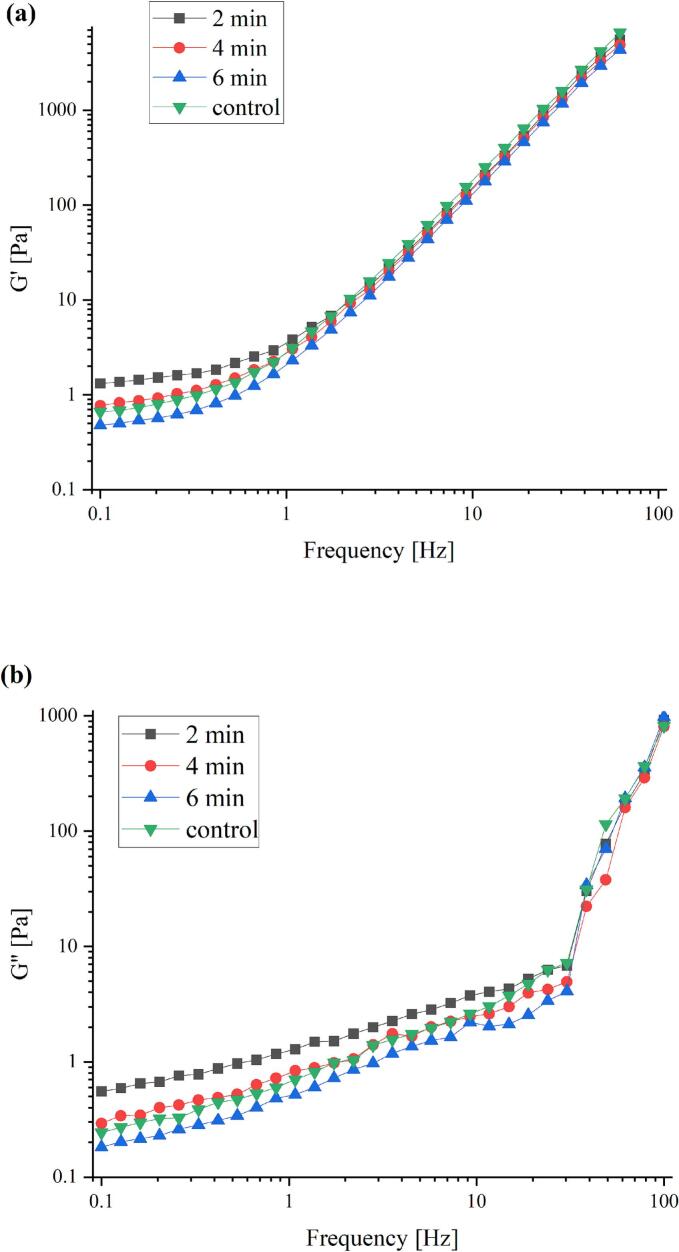
Dynamic storage modulus (a), and loss modulus (b) of control, and plasma-treated complex of soy protein isolate-chia seed gum at different times (2, 4, and 6 min).

One common oscillatory mode test that shows variations in the elastic and viscous portions of materials under constant strain is the frequency sweep test. It is regarded as an effective technique for comparing how treatments affect the viscoelastic properties of materials (Amirabadi et al., 2021). The storage modulus (G') and loss modulus (G") values in both native and plasma-treated SPI-CSG complex solutions are shown in Fig. 6a and b. All the solutions exhibited higher G' and lower G" values with no crossing-over between them. Both moduli were frequency-dependent (0.1–100 Hz), and their values increased with higher frequencies, indicating the formation of a gel-like network structure with weak gel properties. The highest magnitudes of G′ belonged to the 2 min treated solution, and its value at low frequency decreased with increasing treatment time up to 6 min. In general, solutions with higher G′ values exhibit stronger electrostatic interactions among complex particles, which may lead to the creation of a dense interfacial network (X. Zhang et al., 2020).

Misra, Kaur, et al. (2015) observed a similar trend in the alterations of G′ and G′′ values of wheat flour following CAP treatment. Voltage and treatment time affected the variations in G′ values of treated wheat flour. Treating weak flour for 50 min at 70 kV increased G′, but longer treatment times led to a decrease in both moduli. Indeed, the interaction of reactive species with wheat flour leads to these physicochemical changes. The research by Amirabadi et al. (2021) revealed that applying CAP treatment strengthened the elastic properties of treated gum dispersions. The most effective treatment time in this case is 20 min. Analysis across the tested frequency range indicated that all treated gum dispersions exhibited higher G′ values, in which the 20 min CAP-treated gum displayed the greatest enhancement. However, as the treatment time was extended to 40 and 60 min, the G′ value decreased.

### 3.6 Antioxidant activity

Antioxidant activities of plasma-treated SPI-CSG complex solutions at different exposure times are given in Table 1. Treated solutions showed a positive effect in increasing antioxidant activity compared to the untreated solutions. Reactive oxygen and nitrogen species generated during the treatment process can lead to degradation and lower molecular weight in the SPI-CSG structure, resulting in more movement and activity of such molecules and a higher ability to scavenge free radicals (Sahraeian et al., 2023). As the plasma exposure time increases, more active species are generated, resulting in a decrease in the molecular weight of the SPI-CSG. Lower molecular weight can provide more hydrogen to scavenge DPPH radicals (Ahmadian et al., 2023; Wu et al., 2018). The results of Gong et al. (2023) showed an increase in antioxidant activity (DPPH and ABTS tests) in the cold plasma-treated soy protein isolates- proanthocyanidins complexes compared to the untreated sample.

## 4 Conclusion

Cold plasma treatment is a rapid, low-cost, and environmentally friendly method that can be easily used to modify food biopolymers to improve their potential uses. This work offers an approach to employing cold plasma to modify the structures of the SPI-CSG complex and improve its functions. Results of the rheological analysis indicated that plasma treatment duration affected the apparent viscosity and viscoelastic characteristics of the complexes. With increasing exposure time, both the apparent viscosity and modulus showed a decreasing trend, and all solutions exhibited shear-thinning behavior and an elastic weak gel structure. 2 and 6 min plasma treatment significantly enhanced surface hydrophilicity, while 4 min treatment increased its hydrophobicity. FTIR results revealed that the plasma treatment did not alter the main structure of the complex, but it did cause a change in the peak intensities. Furthermore, the treated solutions demonstrated lower zeta potential, smaller droplets, and a higher antioxidant capacity compared to the native solution. This research showed that cold plasma could be a straightforward and efficient technique for modifying biopolymer complexes, potentially expanding their range of applications. Indeed, cold plasma has the potential to alter the functionality of the SPI-CSG complex solution to get the desired characteristics of a particular food product. However, more research is required to optimize CAP parameters for food biopolymers.

## CRediT authorship contribution statement

**Soheila Ahmadian:** Writing – original draft, Validation, Software, Methodology, Investigation, Formal analysis, Conceptualization. **Farshad Sohbatzadeh:** Writing – review & editing, Resources, Investigation, Conceptualization. **Fatemeh Jamshidi Alashti:** Writing – review & editing, Methodology, Investigation. **Reza Esmaeilzadeh Kenari:** Writing – review & editing, Funding acquisition.

## Declaration of competing interest

The authors declare that they have no known competing financial interests or personal relationships that could have appeared to influence the work reported in this paper.

## Data Availability

Data will be made available on request.
